# Impact of gut Microbiome alteration in Ulcerative Colitis patients on disease severity and outcome

**DOI:** 10.1007/s10238-022-00917-x

**Published:** 2022-11-07

**Authors:** Osama Mohammed Basha, Raghda A. Hafez, Sara Mohamed Salem, Reham H. Anis, Amr Shaaban Hanafy

**Affiliations:** 1 Internal Medicine Department, Hepatogastroenterology Unit, Faculty of Medicine, Zagazig University, 40- Mostafa Fouad Street, Sharkia, Zagazig, 44519 Egypt; 2 Medical Microbiology and Immunology Department, Faculty of Medicine, Zagazig University, Zagazig, Egypt

**Keywords:** Ulcerative colitis, Severity, Outcome, microbiota

## Abstract

**Background:**

Ulcerative colitis is a heterogeneous disease in terms of disease course, location, and therapeutic response. The current study was done to assess the alteration of the gut microbiome in UC patients and its relationship to severity, response to therapy, and outcome.

**Patients and methods:**

The study included 96 participants who were divided into a case group (*n* = 48, recent onset, treatment naive ulcerative colitis patients who were subdivided into mild, moderate, and severe subgroups based on Truelove–Witts and endoscopic severity) and a healthy control group (*n* = 48). All were subjected to a thorough history, clinical examination, colonoscopy, routine laboratory tests, and quantitative real-time PCR to quantify Bacteroides, Lactobacilli, Faecalibacterium prausnitzii, Veillonella, and Hemophilus in fecal samples at baseline and 6 months after treatment.

**Results:**

Bacterial 16S rRNA gene sequencing revealed a significant reduction in the phylum Firmicutes in UC patients, with a significant predominance of the phylum Bacteriodetes. F. prausnitzii and lactobacilli were inversely proportional to disease severity, whereas Bacteroides, Hemophilus, and Veillonella were directly proportional to it. Six months after therapy, a statistically significant increase in F. prausnitzii and lactobacilli was observed, with a decrease in the levels of other bacteria. Lower baseline F. praustinizii (< 8.5) increased the risk of relapse; however, lower ESR (< 10), lower post-treatment CRP (< 6), lower Bacteroides (< 10.6) indefinitely protect against relapse.

**Conclusion:**

The gut microbiome of recently diagnosed UC showed lower levels of Lactobacilli, Faecalibacterium, and higher levels of Bacteroides and Veillonella, and the change in their levels can be used to predict response to therapy.

## Introduction

Ulcerative colitis (UC) is an idiopathic, chronic inflammatory disease of the large intestine, frequently involving the rectum, and characterized by continuous inflammation and ulceration of intestinal mucosa and submucosa. This disease causes significant morbidity with an increasing prevalence all over the world. In the USA, UC affects approximately 500,000 individuals with an incidence of 8–12 per 100,000 populations per year and the incidence has remained relatively constant over the last five decades [1].

Crohn’s disease (CD) and UC are two forms of inflammatory bowel diseases (IBD), and while CD can impact any segment of the gastrointestinal tract, UC pathology is restricted to the colon. The precise etiology of UC remains unknown, but factors such as the host immune system, other genetic factors, and environmental factors, contribute to the occurrence of UC. Typical symptoms of UC include abdominal cramping, rectal bleeding, and persistent bloody diarrhea, and other symptoms such as severe fecal urgency resulting from reduced rectal compliance, irritability, general malaise, incontinence, and weight loss are also common [2].

Ulcerative colitis is treated with azathioprine, mesalamine, glucocorticoids, and anti-tumor necrosis factor agents (infliximab and adalimumab, golimumab), α_4_β_7_ integrin blockers as vedolizumab or Janus kinase inhibitor tofacitinib. Therefore, there was a critical need to look into a potential therapeutic target involving the gut microbiome in UC given the high costs of the given drugs, their unexpected toxicities, and the need for meticulous follow-up after their administration, as well as the fluctuating course of the disease; this is especially true given the established knowledge of dysbiosis contribution to the pathogenesis of UC [3].

The gastrointestinal tract serves as a transitory interphase (up to 200 m^2^) between the outer environment and the body with a complex polymicrobial ecology that interacts with internal and external antigens and has a significant impact on health and disease [4].

The gut microbiome is defined as the total collection of microorganisms, bacteria, viruses, protozoa, and fungi, as well as their collective genetic material, that reside in the gastrointestinal tract, some of which are commensal, while others are potentially pathogenic, leading to a possible beneficial relationship [5]

Under healthy states, gut microbiota are non-pathogenic and live in symbiosis with gut enterocytes, thus enhancing gut integrity, intestinal epithelium vitality, energy production, and construction of the immune system memory against many pathogens [6].

Disrupting these beneficial functions has been linked to a wide range of gastrointestinal diseases, including inflammatory bowel disease, irritable bowel syndrome, and hepatocellular carcinoma, metabolic diseases such as obesity and diabetes mellitus, atherosclerosis, non-alcoholic fatty liver disease [7], allergic diseases, and neuropsychological illnesses such as autism, depression, and schizophrenia [8].

An intestinal barrier separates the luminal contents from the underlying immune compartments [9] and specialized secretory cells such as plasma cells, goblet cells, and Paneth cells that secrete IgA, mucus, and antimicrobial proteins that make up the main components of the intestinal mucosa [10] and maintain intestinal homeostasis integrity.

The role of the gut microbiota in the pathogenesis of UC remains to be clarified, microbiota acting on dendritic cells (DC) by secreting substances such as polysaccharide antigen, butyrate, and short-chain fatty acids (SCFAs); DC cells then act on regulatory T (Treg) cells to inhibit inflammation through production of IL-10, transforming growth factor B (TGF-ß) [9, 10].

Reduction in butyrate-producing bacteria as Fecalbacterium Prausnitzii, which is an energy source for intestinal epithelial cells, combined with an increase in sulfate-reducing bacteria (SRB) [11], which metabolize sulfate into hydrogen sulfide, thus blocking butyrate utilization and inhibiting pathogen phagocytosis, increasing colonic epithelial permeability and bacterial translocation [11]; at the same time, Toll-like receptor (TLR), nuclear factor KB production, and inflammatory cytokines such as IL-1b, TNF, IL17, IL21, and IL22, were stimulated, perpetuating mucosal inflammation [12].

A healthy gastrointestinal tract has a low oxygen level and a large population of Firmicutes, which are obligate anaerobes. However, in dysbiosis, a disruption in the anaerobic environment of the gut is seen [13].

Cytotoxic T lymphocyte-associated antigen-4 (CTLA-4) inhibits the signal transduction of T lymphocytes in the presence of antigen-presenting cells and is a key player in the development of immunological tolerance. Its downregulation has been linked to autoimmune and lymphoproliferative disorders. The efficiency of therapy and multi-drug resistance in cancer is significantly influenced by multi-drug resistance 1 (MDR1) [14]. Single-nucleotide polymorphisms in the MDR1 gene, namely rs1045642 C > T, and CTLA-4 gene, primarily rs3087243 G > A and rs231775 G > A, have also been linked to an increased risk of UC [14, 15]. Also CTLA-4 is an inhibitory immune checkpoint that can be accentuated in tumor-infiltrating lymphocytes and colorectal cancer (CRC) cells, facilitating tumor growth and metastasis [16]; in addition, anticancer immunotherapy by CTLA-4 blockade is accentuated by outgrowth of Bacteroides fragilis with its anticancer properties [17].

The current study aimed at determining the relationship between the severity of UC and the changes in gut microbiome composition during the course of the disease, as well as whether these changes could affect disease outcome and response to therapy.

## Methods

### Patients

During the study, 124 patients were evaluated; 76 patients were excluded due to infectious etiology (*n* = 20), Crohn's disease (*n* = 2), recent drug intake (*n* = 29), refusal of endoscopy (*n* = 25). Finally, 48 new-onset treatment-naïve adult ulcerative colitis patients were enrolled in the study as a case group. Patients were included if UC was confirmed by clinical picture, laboratory, colonoscopic, and histological findings. Clinical severity of active UC was evaluated by Truelove and Witts classification. Disease severity was determined based on colonoscopic findings using the Mayo Clinic subscore.

Patients who refused to participate in the study or to undergo colonoscopy, other forms of IBD such as Crohn's disease, acute infectious colitis, history of chronic NSAIDs, antibiotics, or oral corticosteroid intake in the previous 3 months, pregnancy, and lactation were excluded.

The patients were classified according to severity into two subgroups; (Subgroup A) included patients with mild to moderate inflammation, and (Subgroup B) included patients with severe inflammation. They have received treatment according to guidelines and followed up for 6 months. Remission of UC is defined as stool frequency < 3/day with no bleeding or urgency. Relapse is defined as a flare of symptoms in patients who are in clinical remission [18].

### Intervention

All the patients were subjected to full history taking and a thorough physical examination.

#### Laboratory analysis

- Fresh stool sample was tested for visible blood and mucus within 1 h of collection; red and white blood cells were counted and expressed as the mean of categories 0, 1–10, 11–20, 21–50, and > 50 per HPF.
- A complete blood count was performed. The neutrophil lymphocyte ratio (NLR) was detected with a cutoff value of > 2. The MPV was detected within 1 h in order to reduce the swelling of platelets (*n* = 7.8–11.0 fl).
- C-reactive protein (CRP), erythrocyte sedimentation rate 1st hour (ESR 1st h) and 2nd hour (ESR 2nd h), liver function tests, kidney function tests.
- Fecal calprotectin was detected by the enzyme linked immunosorbent assay (ELISA) according to the manufacturer’s instructions (human CALPRO ELISA kit, Sunnyvale, CA, USA). Values up to 50 ug/gm of stool were normal.
- Abdominal imaging as abdominal X-ray in severe UC and abdominal ultrasonography were performed to exclude other causes of abdominal pain.

#### Colonoscopy

The Mayo Clinic subscore system was used to assess the endoscopic severity of UC. Score 0: normal or inactive disease, score 1: mild (erythema, decreased vascular pattern, mild friability), score 2: moderate (marked erythema, absent vascular pattern), and score 3: severe (ulceration with spontaneous bleeding) [19]. For the initial diagnosis of UC, multiple biopsies (at least 2) were taken from five sites around the colon, including the rectum and ileum.

#### Assessment of the fecal microbiome

Approximately 10 g of fresh stool samples was obtained from each subject. Fecal samples were collected again 6 months after treatment. All samples were kept at − 20 °C until they were used.

Genomic DNA was extracted from fecal samples using a QIAGEN stool kit (QIAGEN, Hilden, Germany) from 200 mg of feces following the manufacturer’s instructions.

Amplification was done by conventional PCR to check primer specificity which was performed using the recommended thermal cycling conditions on the Bio-Rad PCR machine (Bio-Rad, USA).

Primers were purchased from operon, Invitrogen. PCRs consisted of 35 cycles, with an initial DNA denaturation at 95 °C (30 s), followed by gradient annealing temperature (30 s) and elongation at 72 °C (45 s). The procedure was completed with a final elongation step at 72 °C (10 min). PCR products were identified using agarose gel electrophoresis.

#### Quantitative real-time PCR

Quantification of gene copies of Bacteroides, Lactobacilli, Faecalibacterium, Veillonella, and Hemophilus groups was carried out for each sample using the ROCHE LightCycler® 480 instrument (Sydney, Australia).

Each PCR was carried out in a final volume of 10 μl, including template DNA, primers, and SYBR® Green PCR master mixture. Thermal cycling conditions started with a reaction cycle at 95 °C for 30 s, followed by 40 cycles of initial denaturation at 95 °C for 5 s and 20 s of annealing at 60 °C.

Standard curves made from known concentrations of plasmid DNA containing the respective amplification for each set of primers were used for quantitative analysis. For further statistical analysis, the numbers were converted to log10 for quantitative analysis.

### Control

Forty-eight healthy participants of matched sex and age served as the control group for comparison.

### Outcome

The study will investigate the relationship between the severity of UC, the disease extent, the response to therapy, and the changes in gut microbiome composition.

### Time frame

A case–control study was carried out over a 12-month period, from December 2020 to December 2021. The research was carried out at Zagazig University Hospital—Faculty of Medicine—Gastroenterology and Hepatology Unit, Medical Microbiology and Immunology Departments. The study was approved by the Institutional Review Board (IRB) of Zagazig University's Faculty of Medicine (IRB reference number: ZUIRB# 6942/2020). The study protocol conforms to the ethical guidelines of the 1975 Declaration of Helsinki and its later amendments.

### Statistical analysis

Data were collected and analyzed using the Statistical Package for the Social Sciences (SPSS) version 20 software. Continuous data were checked for normality by using Kolmogorov–Smirnov test. Values with normal distribution were expressed as mean ± SD; however, non-normally distributed variables were expressed as median (interquartile range). Categorical variables were expressed as frequency and percentage and analyzed using the *χ*2 test or Fisher exact test, and continuous variables were analyzed using the Student’s t test or Mann–Whitney test. If the cell counts were small, the Wilcoxon signed rank test, ANOVA, or Kruskal–Wallis was used appropriately. Multivariable logistic regression was used to detect independent variables of the outcome. *P* value was set at < 0.05 for significant results.

## Results

The study had included 48 patients with new-onset, treatment-naïve adult UC patients who were compared to a group of healthy control subjects (*n* = 48). There was a statistically non-significant difference between the studied groups regarding age or gender distribution. Females made up 41.7%. Age was non-significantly higher in the case group (*P* = 0.06) (Table 1).

**Table 1:** Comparison between baseline demographic and laboratory data in the studied groups

Parameter	Groups		Test
	Case group	Control group	*P*
Male	28 (58.3%)	24 (50%)	0.413
Female	20 (41.7%)	24 (50%)	
Age	36.63 ± 11.01	32 ± 9.25	0.028
Hemoglobin (gm/dl)	9.54 ± 1.34	13.52 ± 0.65	<0.0001
WBCs (10^3^/µl)	9.93 ± 2.25	8.13 ± 1.51	<0.0001
N/L ratio	3.69 ± 0.95	2.03 ± 0.24	<0.0001
Platelet (10^3^/µl)	385.7 ± 67.6	383.3 ± 62.6	0.857
MPV (fl)	8.61 ± 1.08	10.22 ± 0.8	<0.0001
ESR (mm/h)	49.75 ± 21.29	7.25 ± 2.21	<0.0001
CRP (mg/l)	7.5 ± 0.95	2.4 ± 1.1	<0.0001
Stool WBC’s (cell/HPF)	58.96 ± 31.3	4.2 ± 1.2	<0.0001
Stool RBC’s (cell/HPF)	50.5 ± 16.2	3.5 ± 1.4	<0.0001
Stool calprotectin ug/gm	952.5 ± 330.3	18.4 ± 9.2	<0.0001

The mean hemoglobin and MPV were significantly lower in the newly diagnosed UC group (*P*  <0.0001 and <0.0001, respectively), while WBC's count and NLR were significantly higher in the UC group (*P*  <0.0001). The baseline mean values of ESR1st h, CRP, stool WBCs, RBCs, and stool calprotectin were significantly higher in the UC group when compared to the healthy control group (*P* < 0.0001) (Table 1).

Left side colitis was evident in 37.5%, proctosigmoid affection in 29.2%, while pan-colitis occurred in 33.3% (*P* = 0.687). According to Truelove–Witts grading and endoscopic grade of severity, 41.7%, 25%, and 33.3% had mild, moderate, and severe grades, respectively (*P* = 0.0386) (Table 2).

**Table 2:** Extent of the disease, degree of severity in the UC patients

	*N* = 48	*p*
*Side*		
Left side	18 (37.5%)	0.687
Proctosigmoid	14 (29.2%)	
Pancolitis	16 (33.3%)	
*Endoscopic severity*		
Mild	20 (41.7%)	0.0386
Moderate	12 (25%)	
Severe	16 (33.3%)	

**Fig. 1:**
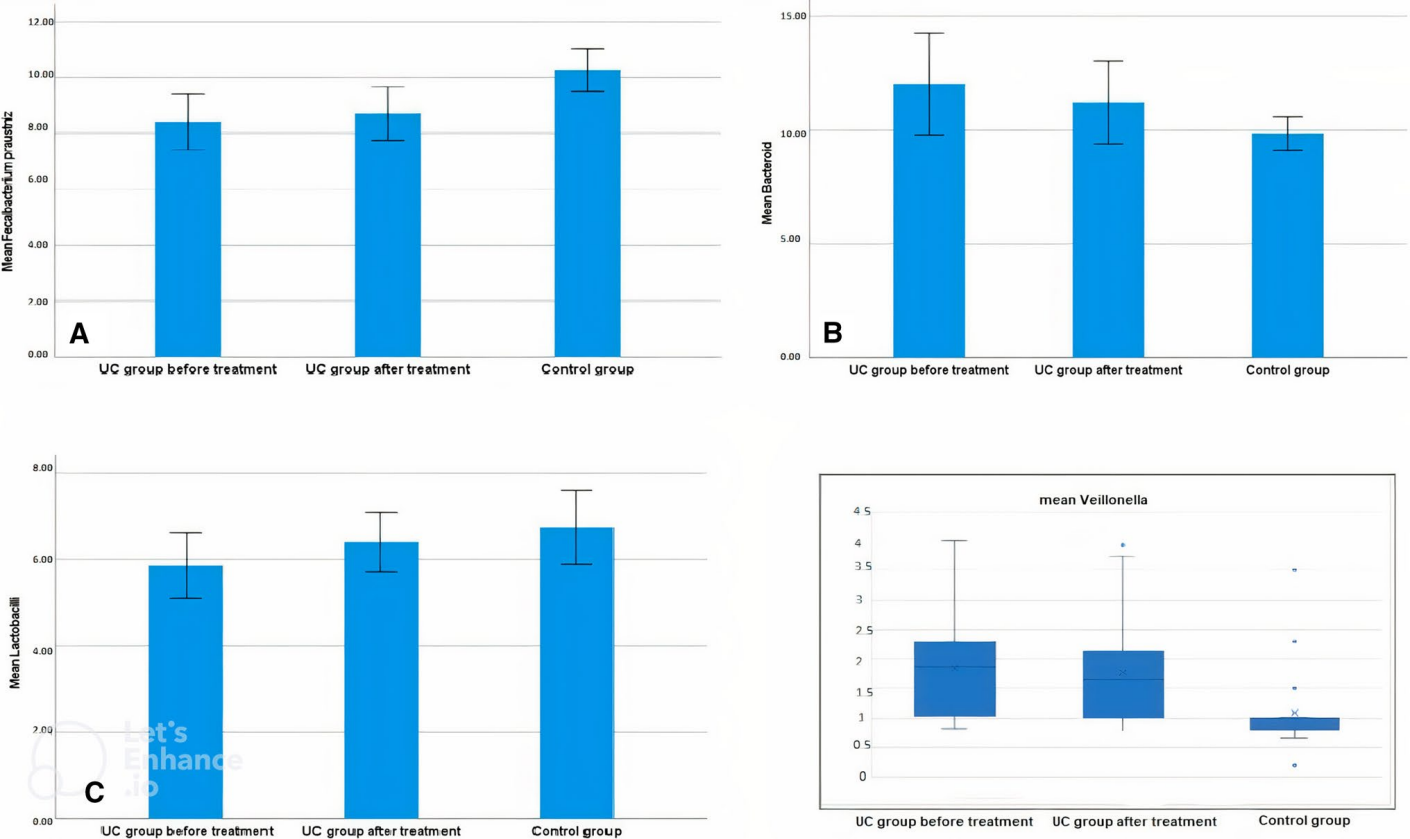
Different microbiota levels in the studied groups. **A** F. prausnitzii count in case group before and after treatment in comparison with the control group. **B** Bacteroides level in case group before and after treatment in comparison with the control group. **C** Lactobacilli in case group before and after treatment in comparison with the control group. **D** Boxplot showing Veillonella count in case group before and after treatment in comparison with the control group

There is a statistically significant difference between the studied case and control groups regarding microbiota; F. prausnitzii and lactobacilli were significantly lower in UC patients (*P* < 0.0001), while both Bacteroides and Veillonella were significantly higher (*P* < 0.0001). Hemophilus was detected in a low level of UC patients, but it was not detected in the control group (Table 3, Fig. 1).

**Table 3:** Comparison between the studied groups regarding gut microbes detected by PCR before treatment

Microbes (log10 copies/g feces)	Group		Test	
	Case group	Control group	*t*	*P*
	Mean ± SD	Mean ± SD		
F. prausnitzii	8.41 ± 1.0	10.27 ± 0.74	10.36	<0.0001
Lactobacilli	5.86 ± 0.76	6.74 + 0.69	5.94	<0.0001**
Bacteroides	12.01 ± 2.25	9.84 ± 0.74	6.35	<0.0001**

	Median (range)	Median (range)	*Z*	*P*
Veillonella	1.86 (0.83–4.02)	1.0 (0.2–3.5)	− 4.031	<0.0001**
Hemophilus	1.87 (0.32–4.06)	Not detected		

Z: Mann–Whitney, t: independent sample *t* test *p < 0.05 is statistically significant **p ≤ 0.001 is statistically highly significant

There is a statistically significant increase in mean values of hemoglobin, serum albumin, and mean platelet volume with a significant decrease in the mean value of white blood cell count, N/L ratio, platelet count, fecal calprotectin, and ESR 6 months after treatment in the UC group (Table 4).

**Table 4:** Laboratory data in case group before and 6 months after treatment

	Pre-treatment	Post-treatment	*t*	*P*
	Mean ± SD	Mean ± SD		
Hemoglobin (gm/dl)	9.54 ± 1.34	10.63 ± 2.15	− 11.447	0.022*
WBCs (10^3^/µl)	9.93 ± 2.25	6.92 ± 2.3	8.953	< 0.001**
N/L ratio	3.69 ± 0.95	2.02 ± 0.16	8.713	< 0.001**
Platelet (10^3^/µl)	385.67 ± 67.63	347.71 ± 28.36	2.348	0.028*
MPV (fl)	8.61 ± 1.08	9.78 ± 0.3	− 4.983	< 0.001**
Albumin (gm/dl)	3.4 ± 0.31	4.38 ± 0.44	− 11.252	< 0.001**
CRP (mg/l)	7.5 (3.5–46)	4.2 (3.2–52)	− 0.8	0.442
Calprotectin ug/gm	952.5 ± 330.3	453.42 ± 168.47	8.7	< 0.001**
ESR (mm/h)	49.75 ± 21.29	10.43 ± 3.79	9.477	< 0.001**
Stool WBCs	58.96 ± 31.31	18.63 ± 17.5	6.289	< 0.001**

t: paired sample t test **p ≤ 0.001 is statistically highly significant

A significant increase in F. prausnitzii and Lactobacilli is associated with a statistically significant decrease in Bacteroides, Veillonella, and Hemophilus 6 months after treatment (Table 5).

**Table 5:** Comparison of gut microbes detected by PCR in case group before and after treatment

Gut microbe (log10/g feces)	Time		Test	
	Pre-treatment	Post-treatment	*t*	*p*
	Mean ± SD	Mean ± SD		
F. prausnitzii	8.41 ± 1.0	8.72 ± 0.97	− 2.35	0.028*
Lactobacilli	5.86 ± 0.76	6.4 ± 0.69	− 4.127	< 0.001**
Bacteroides	12.01 ± 2.25	11.21 ± 1.82	2.793	0.01*

	Median (range)	Median (range)	Wx	p
Veillonella	1.86 (0.83–4.02)	1.65 (0.79–3.92)	− 3.719	< 0.001**
Hemophilus	1.87 (0.32–4.06)	1.43 (0.25–3.72)	− 3.39	< 0.001**

t: paired sample t test **p ≤ 0.001 is statistically highly significant

In the UC group after treatment, although F. prausnitzii, Lactobacilli showed a statistically significant increase from baseline, they were still lower when compared to the control group (*P* < 0.0001, 0.069, respectively). Both Bacteroides and Veillonella were still higher when compared to the control group, despite the significant decrease from baseline mediated by UC treatment (Table 6, Fig. 1).

**Table 6:** Comparison between the studied groups regarding gut microbes detected by PCR after treatment

	Group		Test	
Gut microbe (log 10 copies/gm feces)	Case group after treatment Mean ± SD	Control group Mean ± SD	*t*	*p*
F. prausnitzii	8.72 ± 0.97	10.27 ± 0.74	8.80	< 0.0001**
Lactobacilli	6.4 ± 0.69	6.74 + 0.69	− 1.513	0.069
Bacteroides	11.21 ± 1.82	9.84 ± 0.74	4.79	< 0.0001**

	Median (range)	Median (range)	*Z*	*p*
Veillonella	1.65 (0.79–3.92)	1.0 (0.2–3.5)	− 3.793	< 0.001**
Hemophilus	1.43 (0.25–3.72)	Not detected		

Z: Mann–Whitney test, t: independent sample t test *p < 0.05 is statistically significant **p ≤ 0.001 is statistically highly significant

Clinical and endoscopic remission was achieved in 34/48 patients (70.8%), 6.2 ± 1.5 months after therapy. Treatment-induced remission caused a change in the abundance of microbiota; those in remission had significantly higher F. praustinizii levels and significantly lower lactobacilli level than those who had a relapse. Bacteroides showed a significant decrease after treatment in subgroups with remission or relapse; however, it remained significantly higher in patients who had relapsed. Veillonella was significantly decreased after treatment in those in remission. However, Hemophilus showed higher levels in patients with remission, with a significant decrease in relapsed patients (Table 7).

**Table 7:** Comparison between subgroups classified by outcome regarding gut microbes

Gut microbes (log 10 copies/gm feces)	Response		Test	
	Remission	Relapse	*t*	*p*
	Mean ± SD	Median (range)		
F. praustinizii				
Before	8.7 ± 0.76	7.71 ± 1.23	1.974	0.042*
After	9.14 ± 0.51	7.72 ± 1.13	3.187	0.008*
P (t)	0.006*	0.971		
Lactobacilli				
Before	6.04 ± 0.51	5.42 ± 1.09	1.446	0.095
After	6.56 ± 0.55	6.03 ± 0.88	1.487	0.088
P (Wx)	0.002*	0.111		
Bacteroides				
Before	11.61 ± 2.18	12.99 ± 2.25	− 1.392	0.089
After	10.63 ± 1.32	12.62 ± 2.18	− 2.259	0.027*
P (Wx)	< 0.001**	0.004*		
Veillonella				
Before	1.49 (0.83–4.02)	2.03 (0.95–2.86)	− 0.635	0.525
After	1.42 (0.79–3.92)	1.98 (1–2.76)	− 0.572	0.567
P (Wx)	< 0.001**	0.105		
Hemophilus				
Before	1.42 (0.32–4.06)	3.22 (1.12–3.98)	− 0.859	0.391
After	1.51 (0.25–2.73)	1.43 (1.01–3.72)	− 0.672	0.502
P (Wx)	0.009*	0.018*		

Z Mann–Whitney test, WX Wilcoxon signed rank test **P ≤ 0.001 is statistically highly significant

F. praustinizii and lactobacilli levels were inversely proportional with the extent of disease, being significantly more prevalent in proctosigmoid UC followed by left-sided colitis and pan-colitis. After treatment, they were significantly increased in the pan-colitis subgroup when compared to the pretreatment level.

**Fig. 2:**
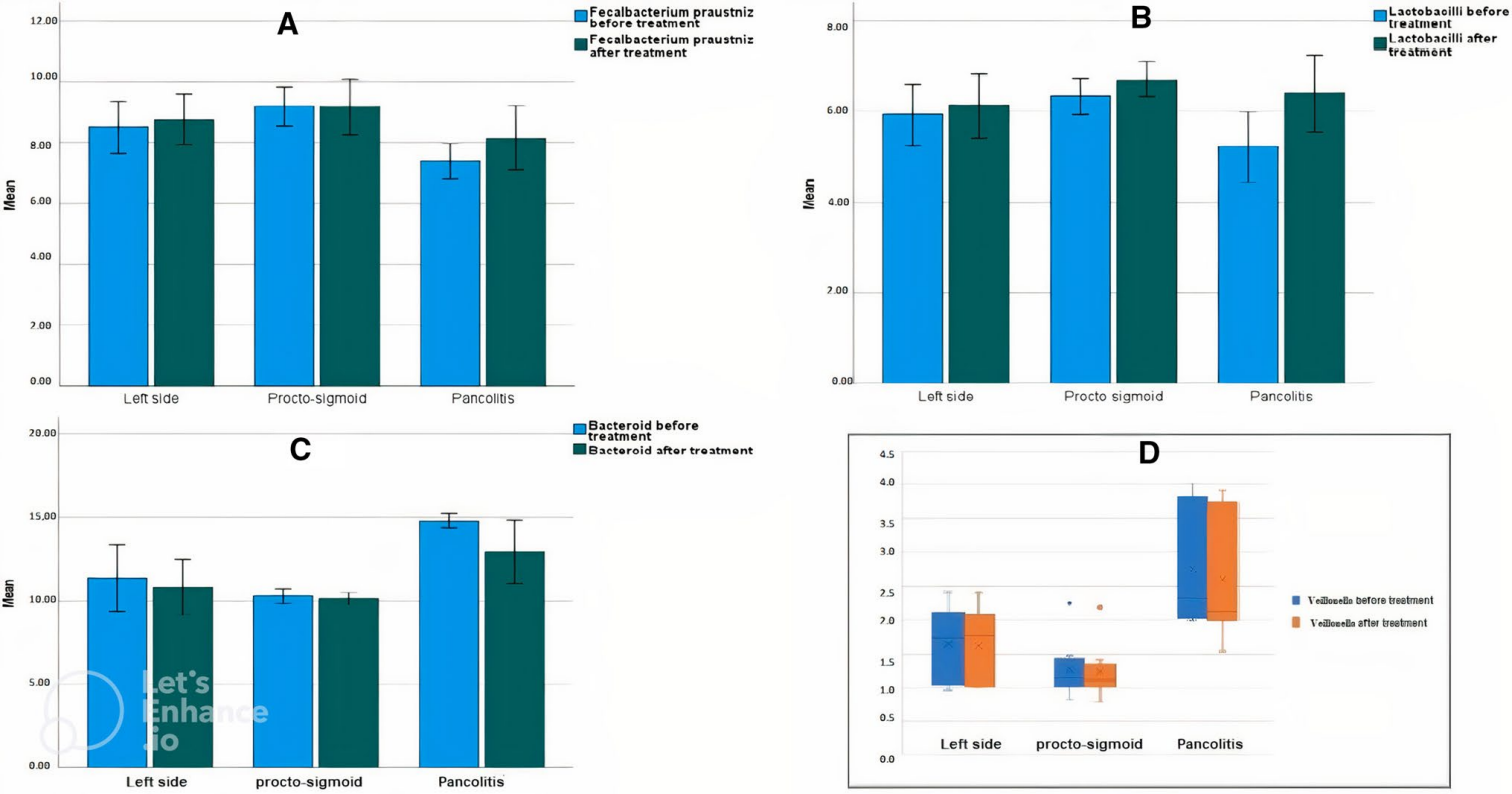
Multiple bar charts showing microbiota levels affected by the site of UC lesions. **A** F. praustinizii before and after treatment according to site of lesion. **B** Lactobacilli before and after treatment according to site of lesion**. C** Bacteroides before and after treatment according to site of lesion**. D** Veillonella before and after treatment according to site of lesion

Bacteroides, Veillonella, and Hemophilus were significantly higher in pancolitis, followed by left-sided and proctosigmoid subgroups, denoting that their level is directly proportional to the extent of the disease. Treatment significantly caused a decrease in their levels in patients with pancolitis (Fig. 2).

Logistic regression analysis revealed that variables associated with disease relapse were lower baseline F. praustinizii (< 8.5 log 10 copies/g feces), but lower ESR (< 10 mm/h), lower post-treatment CRP (< 6 mg/l), lower Bacteroides (< 10.6 log 10 copies/g feces) protected against relapse indefinitely.

## Discussion

Ulcerative colitis is regarded as a polygenic disease with the interplay of multiple etiologies, including environmental, genetic, and immune modulatory factors leading to intestinal mucosal inflammation and ulceration [20, 21].

The gut microenvironment provides a good microbiome habitat which can benefit the host by producing short-chain fatty acids and essential vitamins. Symbiosis refers to the mutual relationship between the host and the gut bacteria [22, 23].

The intestinal microbiome is regarded as a vital organ that has been linked to a variety of gastrointestinal diseases. Because the composition of the intestinal microbiome remains stable over time, many studies have suggested that it is a potential predictor of health status and a potential therapeutic target [24].

According to some studies, the gut microbiome composition varied between active and dormant UC stages. Furthermore, a one-year study of the gut microbiome revealed that the gut microbiome was affected in UC and remained unstable even after remission had been achieved and that could be a hot point of research for a possible therapeutic target [25].

Because many patients have an incomplete response to treatment, assessing progression risk and determining optimal therapy for ulcerative colitis are difficult. Microbial taxonomic composition was examined from fecal specimens and showed a depletion of the core gut microbiome and expansion of bacteria typical for the oral cavity (Veillonella, Hemophilus) which were associated with disease activity [26]. Potentially pathogenic gut microbiota can act through expanding pro-inflammatory species and restriction of protective species [27].

In the current study, based on bacterial 16S rRNA gene sequencing, we discovered a significant decrease in the phylum Firmicutes in UC patients, while the phylum Bacteriodetes was predominating. At the genus level, there was a significant decrease in the short-chain fatty acid producer F. prausnitzii, lactobacilli with a significant predominance of Bacteroides, and Veillonella, despite the fact that the latter belongs to Firmicutes and is an oral cavity resident whose transition to the colon may initiate UC [28].

A longitudinal study enrolled 51 patients with UC, 24 of whom were in remission and 27 of whom had active UC at the time of enrollment. Seven of the 24 remission patients developed relapse and showed lower diversity, with a higher proportion of Bacteroides (*P* = 0.047) [27]. Indeed, bacterial infection-driven dysbiosis and environmental factors had been linked to IBD through inducing an imbalance with a shortage of mucosal protective bacteria such as F. prausnitzii [29].

In a study of UC patients, the dominant bacterial families were Veillonellaceae and Ruminococcaceae, accounting for 15.8% and 14%, respectively, and were associated with a decrease in Faecalibacterium and Bifidobacterium [30].

The decrease in F. prausnitzii in UC compared to controls supported its potentially protective role; it is one of the main butyrate-producing microbiota in the gut, which likely contributes to its anti-inflammatory activity [31], via the production of an anti-inflammatory protein (15 kDa) that inhibits the NF-B pathway in intestinal epithelial cells [32], which was consistent with our findings.

Lactobacilli are thought to benefit the host, and numerous studies have shown that certain lactobacilli strains can reduce the severity of UC and keep it in remission [33]. Lactobacilli were found to be significantly lower in the mucosa of inflammatory bowel disease patients [34]. Lactobacilli levels were significantly lower in the UC group in the current study, with a significant increase after 6 months of treatment.

Bacteroides and Veillonella were significantly higher in UC patients prior to treatment initiation (*P* = 0.001), but they showed a statistically significant decrease 6 months later, which was supported previous studies that reported an increase in Veillonella in the UC patient group [35, 36]. A study examined the mucosal tissue samples from patients with active UC compared to healthy controls which revealed a higher incidence of populations of members of the Bacteriodetes in UC [37].

A comparison of the microbiological composition of the intestines of ulcerative colitis patients and healthy people revealed that Hemophilus was 20.5 times more prevalent (*P* = 0.01) [38]. In UC biofilms, opportunistic pathogens such as Hemophilus influenza were found [39]. Patients with initially severe disease had higher levels of H. influenza at baseline, which gradually decreased with treatment, indicating that a decrease in H. influenza abundance may be associated with improved disease outcome [26], which was consistent with our findings that Hemophilus was detected in UC patients and was significantly reduced 6 months after therapy. 

In the current study, it was shown that successful UC treatment had a positive impact on the presence of beneficial microbiota, as there was a statistically significant increase in F. prausnitzii and Lactobacilli 6 months after treatment, which was supported by previous studies; corticosteroid therapy or infliximab could completely restore F. prausnitzii levels [40], and even fecal microbiota transplantation, a potential therapy for modulating gut microbiota, had enhanced the colonization of F. prausnitzii and Bifidobacterium [41].

The current study demonstrated for the first time that levels of beneficial microbiota F. praustinizii and lactobacilli were inversely proportional to the extent of disease affection and severity of UC; the shorter the colonic area affected, the higher the prevalence of beneficial microbiota, suggesting that restoring their levels in severe cases may predict successful response to therapy. Bacteroides, Veillonella, and Hemophilus levels, on the other hand, were directly proportional to the extent of the disease and its clinical severity, and treatment significantly reduced their levels, particularly in patients with pan-colitis; as a result, the course and extent of UC could be predicted by the degree of abundance of these bacteria.

The limitations of the current study were that it was a single-center study. A more precision and individualized approaches need to be tested in UC to confirm gut dysbiosis as indirect fecal markers, for example fecal short-chain fatty acids as acetate, butyrate, or propionate. We did not extend examination to UC patients who developed colorectal cancer to study the pattern of microbiota in these patients and cases with CRC and dysbiosis should be investigated for tumoral CTLA-4 expression to initiate a predictive analysis for treatment response before using capecitabine which can downregulate CTLA-4 [16]; these points could be hot topics for future research and are beyond the scope of the manuscript.

In conclusion, in naive patients with newly discovered UC, the levels of potentially pathogenic gut microbiota such as Bacteroides, Veillonella, and Hemophilus were directly proportional to the extent of the disease and clinical severity, and treatment significantly reduced their levels while improving the levels of beneficial microbiota such as F. lactobacilli and prausnitzii.

## Data Availability

Data cannot be shared for confidentiality reasons. Queries about the data should be directed to the corresponding author.
